# Multisite Phosphorylation of the Guanine Nucleotide Exchange Factor Cdc24 during Yeast Cell Polarization

**DOI:** 10.1371/journal.pone.0006563

**Published:** 2009-08-10

**Authors:** Stephanie C. Wai, Scott A. Gerber, Rong Li

**Affiliations:** 1 Stowers Institute for Medical Research, Kansas City, Missouri, United States of America; 2 Biological and Biomedical Sciences Graduate Program, Harvard Medical School, Boston, Massachusetts, United States of America; 3 Department of Cell Biology, Harvard Medical School, Boston, Massachusetts, United States of America; 4 Department of Molecular and Integrative Physiology, University of Kansas Medical Center, Kansas City, Kansas, United States of America; University of Birmingham, United Kingdom

## Abstract

**Background:**

Cell polarization is essential for processes such as cell migration and asymmetric cell division. A common regulator of cell polarization in most eukaryotic cells is the conserved Rho GTPase, Cdc42. In budding yeast, Cdc42 is activated by a single guanine nucleotide exchange factor, Cdc24. The mechanistic details of Cdc24 activation at the onset of yeast cell polarization are unclear. Previous studies have suggested an important role for phosphorylation of Cdc24, which may regulate activity or function of the protein, representing a key step in the symmetry breaking process.

**Methodology/Principal Findings:**

Here, we directly ask whether multisite phosphorylation of Cdc24 plays a role in its regulation. We identify through mass spectrometry analysis over thirty putative *in vivo* phosphorylation sites. We first focus on sites matching consensus sequences for cyclin-dependent and p21-activated kinases, two kinase families that have been previously shown to phosphorylate Cdc24. Through site-directed mutagenesis, yeast genetics, and light and fluorescence microscopy, we show that nonphosphorylatable mutations of these consensus sites do not lead to any detectable consequences on growth rate, morphology, kinetics of polarization, or localization of the mutant protein. We do, however, observe a change in the mobility shift of mutant Cdc24 proteins on SDS-PAGE, suggesting that we have indeed perturbed its phosphorylation. Finally, we show that mutation of all identified phosphorylation sites does not cause observable defects in growth rate or morphology.

**Conclusions/Significance:**

We conclude that lack of phosphorylation on Cdc24 has no overt functional consequences in budding yeast. Yeast cell polarization may be more tightly regulated by inactivation of Cdc42 by GTPase activating proteins or by alternative methods of Cdc24 regulation, such as conformational changes or oligomerization.

## Introduction

Cell polarization is the process by which cells establish asymmetry along a single axis and is essential for processes such as cell migration and asymmetric cell division [1]. Budding yeast is an excellent model for the study of cell polarity, because it divides asymmetrically between the mother and bud and displays a characteristic cell and actin morphology at each cell cycle stage, facilitating the study of different polarity states [2]. Also, many proteins involved in cell polarity, such as the Rho GTPase Cdc42, are conserved from yeast to mammals [3], [4], [5]. Yeast cells are round and unpolarized in G1 phase, but after the G1-S transition, the actin cytoskeleton and localization of the Cdc42 GTPase are polarized to the presumptive bud site [2]. The cell is then set up for bud emergence, which occurs shortly afterwards. Bud formation through polarized growth is required for successful cell division.

The Cdc42 GTPase has been shown to regulate cell polarity in many organisms, including budding yeast [6]. Like most GTPases, Cdc42 is active when bound to GTP, and this is generally catalyzed by proteins called guanine nucleotide exchange factors (GEFs). In budding yeast, the only known GEF for Cdc42 is Cdc24. Both genes are essential, and the loss of either results in large unbudded multi-nucleate cells, indicative of an inability to undergo polarized growth [7], [8]. Activated Cdc42 signals through its downstream effectors to assemble and organize the actin cytoskeleton and the secretory machinery [2], [6]. Because Cdc42 is well conserved and important for cell polarity, much work has been done to characterize its function and its downstream effectors. Upstream regulation, however, particularly control of its GEF Cdc24, is not as well understood. Despite several studies, the mechanism of Cdc24 regulation remains unclear. Cdc24 belongs to the conserved family of Dbl-homology (DH) GEFs, which are characterized by adjacent DH and pleckstrin homology (PH) domains [9]. The DH domain is responsible for GEF activity, and the PH domain is thought to help localize or orient the GEF at the plasma membrane. Cdc24 has two other conserved functional domains: an N-terminal calponin-homology (CH) domain, the function of which is unclear, and a C-terminal PB1 domain, which mediates binding of Cdc24 to the adaptor protein Bem1 (see below).

Polarization of yeast cells must occur at the proper time in the cell cycle, suggesting a potential regulatory role for the cyclin-dependent kinase (CDK) Cdc28 on Cdc24, which leads to timely activation of Cdc42. Interestingly, Cdc24 is sequestered in the nucleus during G1 by the CDK-inhibitor Far1 [10], [11]. At bud emergence, Far1 is phosphorylated and degraded, releasing Cdc24 into the cytoplasm. Cdc24 mutants unable to bind to Far1 are constitutively located in the cytoplasm; however, Cdc28 activity is still required to activate these cytoplasmic mutants, indicating that nuclear export of Cdc24 is not sufficient to promote GEF activity [11], [12]. This suggests that Cdc28 directly or indirectly activates Cdc24. Cdc28 has been shown to phosphorylate Cdc24 *in vitro* [13], but it is unclear whether this occurs *in vivo*.

Yeast cell polarity is also regulated in a spatial manner. Haploid yeast cells generally bud adjacent to the previous bud scar, which is a remnant from the previous cell division site. The Ras family GTPase Bud1/Rsr1 recruits Cdc24 to the bud site in order to establish polarized growth [14]. Bud1 may also activate Cdc24 by inducing a conformational change [15]. Interestingly, however, *Δbud1* cells grow and divide at the same rate as wild type cells, although they bud in a random pattern [16]. This suggests that regulation of Cdc24 by Bud1 is not required for polarity *per se*. This may be due to redundant activation events mediated by the adaptor protein Bem1. In addition to possibly causing a conformational change by directly binding Cdc24, Bem1 also mediates the formation of a complex containing GTP-bound Cdc42, Cdc24, and the p21-activated kinase (PAK) Cla4, and enables Cla4 to phosphorylate Cdc24 [12], [17]. Our previous work showed that Bem1 is required for a mechanism of cell polarization that is independent of actin filaments [18]. Bem1 may induce cell polarization by mediating a positive feedback loop whereby Cdc24 is preferentially recruited/activated at the site of Cdc42^GTP^ accumulation [19], [20], [21]. Cla4 phosphorylates Cdc24 in this complex likely at multiple sites; the function is not clear, but one study has suggested that this phosphorylation negatively regulates Cdc24 function [12], [17]. Because multisite phosphorylation has been shown in some cases to confer ultrasensitivity onto cell signaling processes [22], [23], one interesting possibility is that phosphorylation of Cdc24 by Cla4 may allow the Bem1 complex to function in a switch-like manner to induce cell polarization. However, despite all of these interesting ideas, there has been no direct study on the functional importance of this phosphorylation *in vivo*.

There is precedent for regulation of Dbl homology GEFs by phosphorylation [9]. The best characterized example is that of the GEF Vav1. It was shown that mouse Vav1 is regulated by an autoinhibitory fold and phosphorylation of a critical tyrosine residue releases this inhibition, activating Vav1 [24]. However, outside of the conserved DH and PH domains, Dbl homology GEFs are highly divergent, and so it is difficult to predict the exact mechanisms by which each GEF is regulated. In this study, we used mass spectrometry to map a large number of *in vivo* phosphorylation sites on Cdc24. Surprisingly, mutagenesis of these putative phosphorylation sites (including those matching consensus sequences for CDK or PAK family kinases and those not) has not resulted in any observable defects in cell polarization and growth by a variety of assays, suggesting that phosphorylation is largely dispensable for the regulation of Cdc24.

## Results

### Purification of Cdc24-TAP

In order to better understand how Cdc24 may be regulated by multisite phosphorylation and, in particular, how this phosphorylation contributes to the Bem1-dependent and actin-independent cell polarization, we set out to purify Cdc24 from yeast cells in order to identify the *in vivo* phosphorylation sites by mass spectrometry analysis. This was challenging because of the following factors: a) Cdc24 is present in low quantities [25] and initial purifications contained many contaminants; b) the level of phosphorylation of Cdc24 changes during the cell cycle [12], [17]; and c) Cdc24 is only partially soluble in cell lysates [26], [27], [28], [29].

The cell lysis and purification protocols were systematically optimized in order to obtain a sufficient amount of phosphorylated protein for analysis. The purification protocol was modified from the tandem affinity purification (TAP) method [30], [31]. Because the mobility shift of Cdc24 on SDS-PAGE is maximal during bud emergence [12], [32] (Figure S1A and B), cell synchronization and release using *cdc28-13*, a temperature-sensitive allele of *CDC28*, was performed to enrich for small-budded cells, and thus phosphorylated Cdc24. Initially, cell lysis was performed using a French press, but Cdc24 in these lysates was mostly insoluble. Solubility of Cdc24 was increased by lysing frozen yeast cells with a coffee grinder and increasing the salt concentration of the lysis buffer (Figure S1C). Contaminants were decreased by precipitating Cdc24 from the cleared lysate with 40% ammonium sulfate precipitation. Most other proteins remained soluble and could thus be separated. More contaminants were removed by washing the IgG column with a buffer with high salt concentration. In order to shorten the time of the purification and decrease the loss of phosphorylated proteins, the calmodulin resin purification step of the TAP method was omitted. The bulk of the GST-TEV protease after cleavage was instead removed by passing the eluate over glutathione resin. The complete final purification scheme is described in Materials and Methods.

### Cdc24 is phosphorylated on a large number of residues

**Figure 1:**
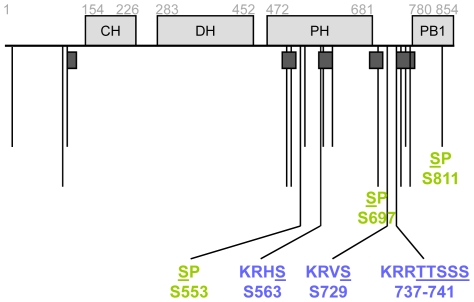
Cdc24 is phosphorylated on 35 residues. Schematic diagram of Cdc24 domain structure, regions of low complexity, and mapped phosphorylation sites. Regions of low complexity are denoted by the dark gray boxes under domains and correspond to residues 106–121, 523–545, 562–600, 680–710, 739–754, and 767–778. Adjacent residues were grouped together to facilitate analysis. Longer lines correspond to more highly ranked sites. Labeled in green are CDK consensus sites and in blue are PAK consensus sites. See Tables S1–S3 for details on sites and ranking information. CH, calponin homology; DH, Dbl homology; PH, pleckstrin homology; PB1, p67^phox^-Bem1 homology.

Cdc24-TAP was purified from 60 g of yeast cells (about 70% were small budded), resolved by SDS-PAGE (Figure S1D), and submitted for mass spectrometry analysis. Mass spectrometry analysis identified 35 residues that were putatively modified by phosphorylation (Figure 1 and Table S1). Most of these residues mapped to the PH domain or the linker region between the PH and PB1 domains. Interestingly, a sequence segmentation program identified sequences in both of these regions as being low-complexity or compositionally-biased [33], [34]. It is tempting to speculate that these intrinsically serine-rich sequences (39.5% serine by composition, on average) may have become favorable substrates for phosphorylation.

Adjacent residues were grouped together to facilitate analysis (e.g. TTSSS, residues 737–741). These single or clustered sites were then ranked based on their scores on NetPhos 2.0 [35] and Scansite [36] (Figure 1; see Tables S1, S2, S3 for more details). Cdc24 contains six CDK consensus sites (S/T P), three of which were mapped. Three of the most highly ranked phosphorylation sites were identified by Scansite to be PKA/PKC consensus sites; however, studies on various PAKs and their substrates have identified a similar consensus sequence [37], [38], and so it is possible that these are sites of PAK phosphorylation.

### Mutation of consensus CDK or PAK sites on Cdc24 does not affect cell growth or morphology

We next used mutagenesis to study the *in vivo* function of the phosphorylation sites in Cdc24. Because of the large number of sites and because of previous work implicating CDK and PAK in Cdc24 regulation, we first examined the CDK and PAK sites. The different phosphorylation mutants are described in Table 1. We designed a construct that would allow homologous recombination of Cdc24 mutants directly into the endogenous locus, generating mutant strains expressing a phosphorylation mutant of Cdc24 as the sole source of Cdc24 in the cell (see Materials and Methods for details). When assessing whether the point mutations had been successfully introduced, we found that mutations of T134 and S245, the sites closest to the 5′ end of *cdc24*, were lost in the CDK mutants, i.e. they had reverted back to the wild type sequence. S245A was retained in the CDK-A/PAK-A mutant. The other mutant residues were successfully mutated, and these are denoted in Table 1. The lost sites were included later in the 35A mutant (see Table 2).

**Table 1:** Mutation of CDK or PAK consensus sites.

Mutant alias	Mutated residues
CDK-3A	S553A, S697A, S811A
CDK-4A	S553A, S697A, T704A, S811A
PAK-A	S563A, S729A, TTSSS(737–741)AAAAA
CDK-A/PAK-A	S245A, S553A, S563A, S697A, T704A, S729A, TTSSS(737–741)AAAAA, S811A
CDK-DE	S553D, S697D, T704E, S811D
PAK-DE	S563D, S729D, T738E, S741D

**Table 2:** Mutation of remaining sites, including non-consensus sequences.

Mutant alias	Mutated residues
PH-A	All mapped residues within the PH domain
Linker-A	All mapped residues within the linker region and T704A
PH-A/linker-A	All mapped residues within PH and linker regions and T704A
35A	All 35 mapped residues and T134A, S245A, T704A

**Figure 2:**
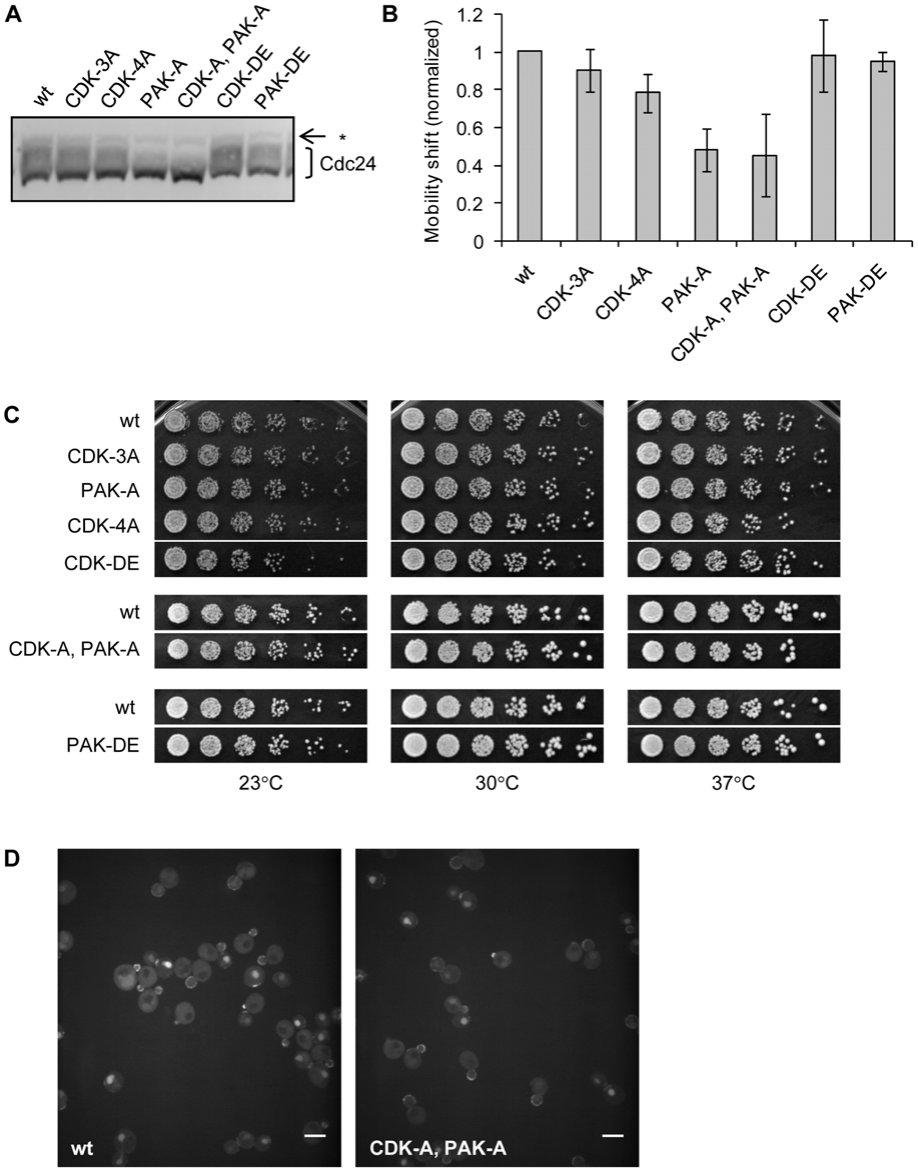
Phosphorylation site mutations decrease mobility shift of Cdc24, but do not affect cell growth or Cdc24 localization. A. Mobility shift of Cdc24 band. Whole cell extracts were prepared from asynchronous cultures of wild type control (wt, RLY2853), *cdc24^CDK-3A^* (RLY2855), *cdc24^PAK-A^* (RLY2858), *cdc24^CDK-4A^* (RLY2934), *cdc24^CDK-DE^* (RLY2938), *cdc24^CDK-A,PAK-A^* (RLY3088), and *cdc24^PAK-DE^* (RLY3089). Blots were probed with rabbit anti-Cdc24 antiserum. Faint band just above smear is a cross-reacting protein (*). B. Quantification and comparison of mobility shift. Mobility shift of band was calculated as (integrated intensity of smear)/(integrated intensity of entire band) and normalized against mobility shift of wild type control resolved on the same gel. Mean and standard deviation of at least three independent samples are shown. C. Serial dilutions of each strain were spotted onto YEPD agar plates and grown for 2 d at 23°C, 30°C, or 37°C, or 13 d at 16°C (not shown). Mutant strains are shown alongside a wild type control that was spotted on the same plate. Only strains relevant to this experiment are shown in this figure. See Materials and Methods for more information. D. Confocal images of strains containing Cdc24-GFP (RLY3096) and Cdc24^CDK-A,PAK-A^-GFP (RLY3099). Scale bar represents 5 µm. Confocal images of other mutants are shown in Figure S2A.

The mutant strains were viable (see below), and we examined whether these mutations affected phosphorylation of Cdc24. Asynchronous cultures of the mutant strains were lysed and resolved by SDS-PAGE to assess the mobility shift of Cdc24, which was shown previously to be caused by phosphorylation [12], [17]. Mobility shift in each mutant strain was quantified and normalized to an appropriate wild type strain resolved on the same gel. Interestingly, a decrease in mobility shift roughly correlated with the increase in number of mutated residues (Figure 2A and B). Mutations of the CDK sites to alanine changed the mobility shift very little, but mutations of the PAK sites to alanine reduced the shift to about 45% of that observed in the wild type. Mutations of predicted phosphorylation sites to aspartate or glutamate did not significantly change the mobility shift. While it is possible that some of these mutations affect the phosphorylation of the Cdc24 protein without changing its mobility shift, it appears that the mutations to alanine, especially of the PAK sites, perturbed Cdc24 phosphorylation.

Because *CDC24* is an essential gene and the only known GEF for Cdc42 in yeast, we expected to see a severe growth phenotype if these mutations affect Cdc24 function. However, these mutations, including the CDK and PAK sites mutated individually or in combination, did not confer cell lethality or temperature-sensitivity, as all strains grew like the wild type control at all temperatures tested (Figure 2C). Cell morphology and budding pattern of the mutants were also similar to the wild type (data not shown). GFP fusions of the mutant Cdc24 proteins localized like the wild type Cdc24, showing the expected localization to the nucleus, presumptive bud site, bud cortex, and bud neck (Figure 2D and S2A). We conclude that these mutations do not grossly affect Cdc24 function or protein localization.

**Figure 3:**
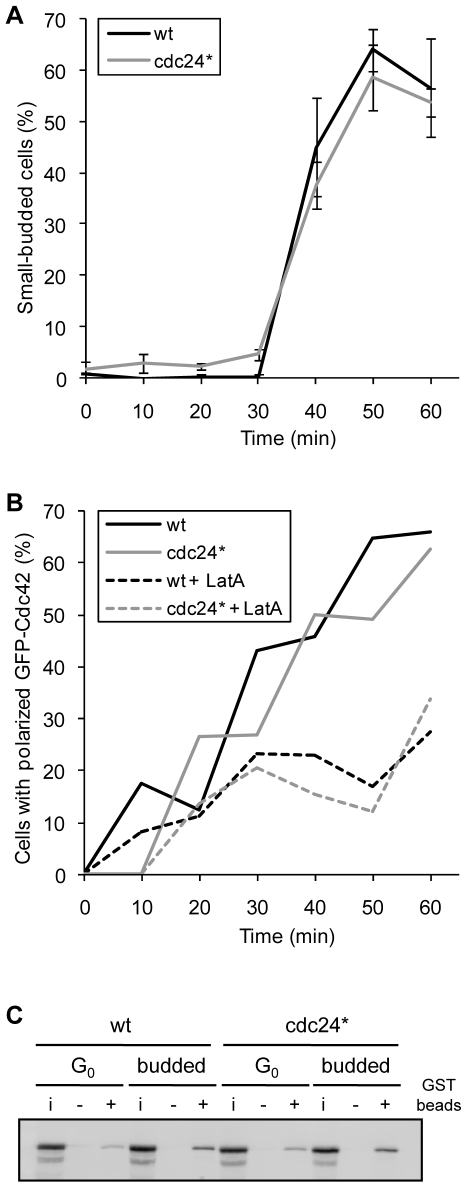
CDK-A/PAK-A mutation does not affect kinetics of bud formation of polarization, nor does it affect overall level of Cdc42 activation. The *cdc24^CDK-A/PAK-A^* strain is labeled here as cdc24* for simplicity. A. Comparison of kinetics of bud formation between wild type (wt, RLY2853) and cdc24* (RLY3088). Cells were arrested with α-factor, then washed and released. Samples of cultures were fixed every 10 min and scored for bud morphology. Percentage of small-budded cells was plotted over time. B. Comparison of kinetics of polarization between wild-type (wt, RLY2903) and cdc24* (RLY3072). Cells were arrested with α-factor, then treated briefly with LatA to depolarize GFP-myc-Cdc42. Cells were then washed and released +/− LatA and scored for polarized localization of GFP-myc-Cdc42. Percentage of cells with polarized GFP-myc-Cdc42 was plotted over time. C. Comparison of levels of active GTP-bound Cdc42 in wild type (wt, RLY2903) and cdc24* (RLY3072). Levels of active Cdc42 were determined at both stationary phase (G_0_) and at the timepoint when most cells had small buds (budded) by measuring levels of GFP-myc-Cdc42 bound to GST-CRIB construct immobilized on glutathione sepharose beads. Small sample of the extract (i), pulldown with GST only beads (−), and pulldown with GST-CRIB beads (+) were resolved by SDS-PAGE. Blots were probed with mouse anti-myc antibody (9E10). Gel shown is representative of two independent experiments.

Because these mutations did not affect cell growth on a gross level, we next asked if the mutations affected Cdc24 function on a subtler level. We focused on the strain containing the most mutations, *cdc24^CDK-A/PAK-A^* (RLY3088). Cell growth assayed by colony formation (as shown in Figure 2C) is at best a rough comparison of growth rates and might not be capable of detecting moderate defects in cell polarity. To this end, we directly assessed budding kinetics in these mutants. Wild type and *cdc24^CDK-A/PAK-A^* mutant cells were arrested in G1 by α-factor and synchronously released into the cell cycle, and the percentage of cells with a small bud was scored at each time point. Budding kinetics of the mutant were comparable to those of the wild type (Figure 3A).

We previously showed that an actin-transport mediated feedback loop works in parallel with a Bem1-dependent polarization pathway [18]. In order to look at the function of the mutant Cdc24 specifically in the Bem1 feedback loop, we abrogated the actin feedback loop by treating the cells with latrunculin A (LatA). The strain was transformed with a plasmid containing GFP-myc-Cdc42 as a marker for polarization. Polarization kinetics of GFP-myc-Cdc42 in the *cdc24^CDK-A/PAK-A^* mutant is similar to that in the wild type, even when cells were treated with LatA (Figure 3B), indicating that Cdc24^CDK-A/PAK-A^ functions normally in the Bem1-dependent cell polarization pathway.

Next, we asked if the CDK-A/PAK-A mutations had any effect on the GEF activity of Cdc24. We used a pull-down assay with the CRIB domain from Cla4, in order to measure the overall level of active GTP-bound Cdc42 in wild type and mutant strain. Both strains were grown to stationary phase, and half of each sample was harvested. The other half was resuspended in fresh media to reenter the cell cycle and harvested when most cells had formed small buds. Beads containing GST-CRIB construct were added to the lysate to pull down GTP-bound Cdc42. As expected, stationary cells contained a lower amount of GTP-bound Cdc42 than small-budded cells. The level of GTP-bound Cdc42 in the *cdc24^CDK-A/PAK-A^* mutant strain was comparable to that in the wild type at both time points (Figure 3C), suggesting that the phosphorylation site mutations do not have a gross impact on Cdc42 activation.

**Figure 4:**
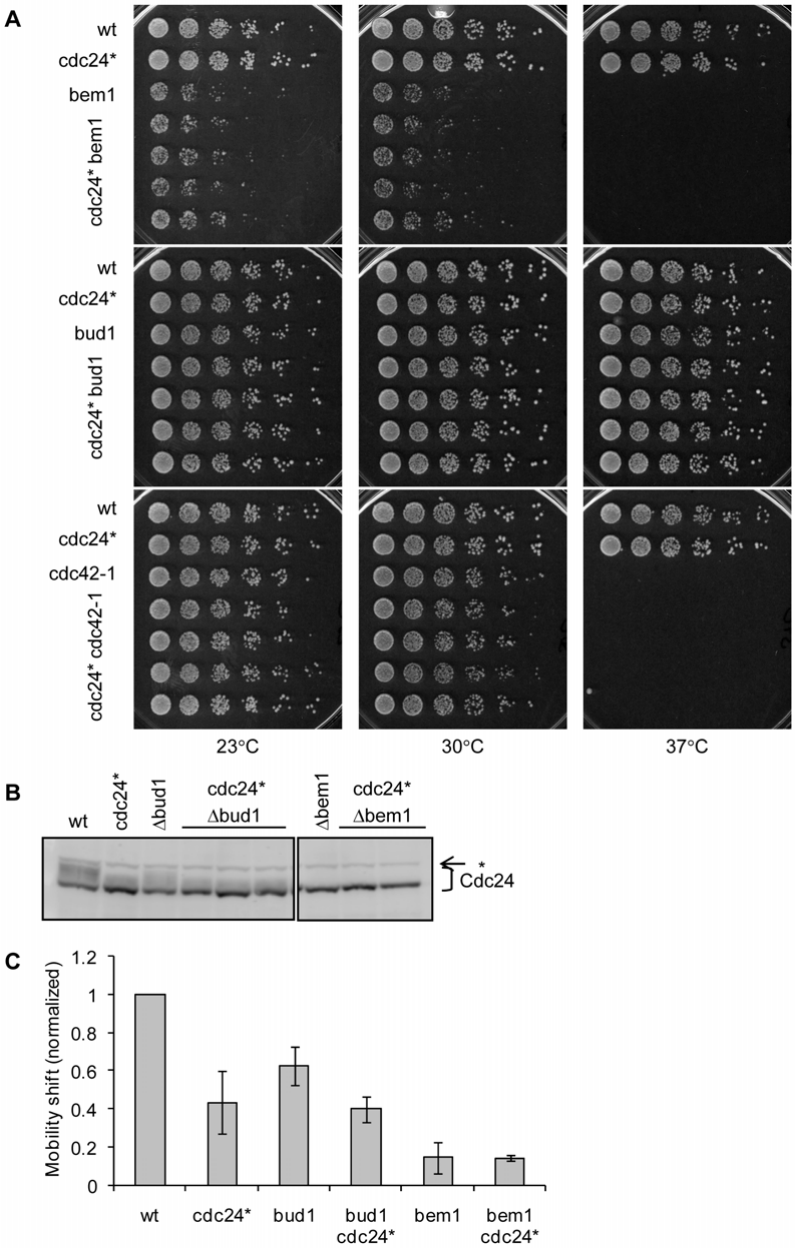
No synthetic effects on cell growth or mobility of Cdc24 are detected between CDK-A/PAK-A mutant and other polarity mutants. The *cdc24^CDK-A/PAK-A^* strain is labeled here as cdc24* for simplicity. A. Serial dilutions of meiotic segregants from crosses between cdc24* (RLY2937) and polarity mutants *Δbem1* (RLY2771), *Δbud1* (RLY2773), and *cdc42-1* (RLY171). Double mutant strains were spotted onto YEPD agar plates and grown for 3 d at 23°C, 30°C, 35°C (not shown), and 37°C. B. Mobility shift of Cdc24 band. Whole cell extracts were prepared from asynchronous cultures of meiotic segregants from crosses between cdc24* (RLY2937) and *Δbem1* (RLY2771) and *Δbud1* (RLY2773). Blots were probed with rabbit anti-Cdc24 antiserum. Faint band just above smear is a cross-reacting protein (*). C. Quantification of mobility shift, calculated, normalized, and displayed as in Figure 2B.

Finally, we examined whether the *cdc24^CDK-A/PAK-A^* mutant displayed any synthetic phenotypes with strains that had defects in polarization or in which the mobility shift of Cdc24 was affected. The *cdc42-1* strain contains a temperature-sensitive allele of *CDC42*, resulting in minor morphological abnormalities at the 23–24°C and growth arrest as unbudded cells at 37°C [8]. The *Δbud1* strain shows no overt polarization defects (apart from a random budding pattern), but we noticed that Cdc24 protein in the *Δbud1* strain had a significantly reduced mobility shift, suggesting an abnormal phosphorylation state (Figure 4). The *Δbem1* strain is viable at 23–30°C, but divides at a much slower rate than wild type cells and displays morphological heterogeneity, with morphological defects such as multiple buds and large unbudded cells [39]. *Δbem1* is nonviable at 37°C. Cdc24 displays little to no mobility shift in the *Δbem1* strain [12], [17].

*cdc24^CDK-A/PAK-A^* was crossed into the *cdc42-1*, *Δbud1*, and *Δbem1* strains, generating strains RLY3093, 3095, and 3102. These diploid strains were sporulated and dissected, and at least eight double mutant spores were analyzed for synthetic phenotypes (Figure 4). None of the double mutants showed temperature-sensitive phenotypes that were different from the single mutants, ruling out a synthetic effect on cell growth. Mobility shift of Cdc24^CDK-A/PAK-A^ in the double mutants was identical to that in the most affected single mutant, ruling out a synthetic effect on phosphorylation (Figure 4). It is interesting to note that wild type Cdc24 in *Δbud1* cells have a less affected mobility shift than Cdc24^CDK-A/PAK-A^ in *Δbud1* cells. This may suggest that phosphorylation sites affected in the CDK-A/PAK-A mutant encompass those that are perturbed in *Δbud1*.

Although we did not explicitly test mating efficiency of *cdc24^CDK-A/PAK-A^*, the strain arrested in α-factor and mated with the *cdc42-1*, *Δbud1*, and *Δbem1* strains with efficiency indistinguishable from that of the wild type strain. Thus, *cdc24^CDK-A/PAK-A^* did not show obvious defects in pheromone response. Taken together, the above results suggest that a lack of phosphorylation on the CDK and PAK consensus sites in Cdc24 has no overt functional consequences, not even when combined with mutations in proteins known to directly interact with Cdc24.

### Mutation of remaining phosphorylation sites on Cdc24 also does not affect cell growth or morphology

Because we were unable to identify a regulatory role for the consensus CDK or PAK sites, we mutated the remaining mapped sites in order to determine if any of these sites may be contributing to Cdc24 regulation. Recent studies have indicated the importance of either the location or the number of phosphorylated sites in the regulation of protein function [23], [32]. Because of the clustering of sites within the PH domain and within the linker region between the PH and PB1 domains, we generated mutants containing alanine mutations of all the mapped residues within either of those domains or in both. We also generated a mutant containing alanine mutations of all 35 mapped residues. In all of these mutants, we included any CDK consensus sites within the region, whether or not they were mapped. Table 2 summarizes these mutants. Because of our earlier difficulty in maintaining mutated sites near 5′ end of *cdc24* by genomic integration, we introduced these mutants via a centromeric plasmid into a strain heterozygous for deletion of *CDC24*. In order to characterize these new *CDC24* mutants, the strains were sporulated and dissected, and spores were identified that carried a genomic deletion of *CDC24*, rescued by the mutant *cdc24* on the plasmid.

**Figure 5:**
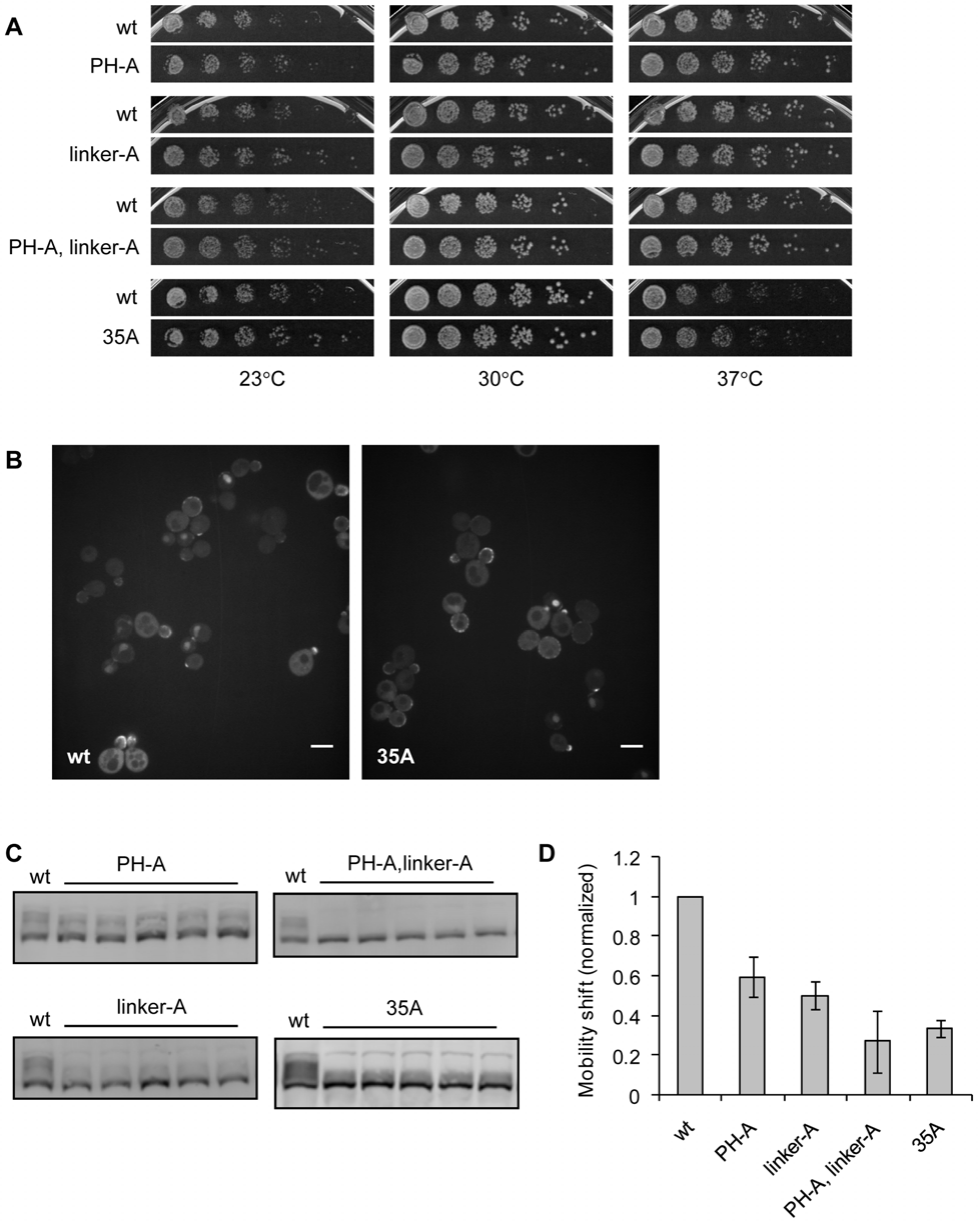
Mutations of non-consensus sites also do not affect cell growth or Cdc24 localization, but they further decrease mobility shift of Cdc24. A. Serial dilutions of wild type control (wt, RLY2530), *cdc24^PH-A^* (RLY3391), *cdc24^linker-A^* (RLY3393), *cdc24^PH-A,linker-A^* (RLY3390), and *cdc24^35A^* (RLY3461) were spotted onto YEPD agar plates and grown for 2 d at 23°C, 30°C, and 37°C, or 7 d at 16°C (not shown). Mutant strains are shown alongside a wild type control that was spotted on the same plate. Only strains relevant to this experiment are shown in this figure. See Materials and Methods for more information. B. Confocal images of strains containing Cdc24-GFP (RLY3437) and Cdc24^35A^-GFP (RLY3468). Confocal images of other mutants are shown in Figure S2B. C. Mobility shift of Cdc24 band. Whole cell extracts were prepared from asynchronous cultures. Blots were probed with rabbit anti-Cdc24 antiserum. Faint band just above smear is a cross-reacting protein (*). D. Quantification of mobility shift, calculated, normalized, and displayed as in Figure 2B.

Like earlier mutants, these mutants did not confer cell lethality or temperature-sensitivity (Figure 5A). Surprisingly, even the mutants containing the most mutations (PH-A/linker-A and 35A) grew like the wild type strain at all temperatures tested. Localization of GFP fusion proteins appeared to be normal and indistinguishable from wild type (Figure 5B and S2B). These results indicate that, like the earlier mutations, these mutations do not grossly affect Cdc24 function or localization in the cell.

We did, however, observe a decrease in the mobility shift of these mutants. Using the procedure described earlier, we quantified the mobility shift of the Cdc24 band from asynchronous cultures resolved by SDS-PAGE (Figure 5C and D). The PH-A/linker-A mutant showed the greatest decrease in mobility shift, retaining on average only 27.2% of the wild-type mobility shift. By comparison, *Δbem1* cells have on average 11.1% of a wild type mobility shift. It is unclear why the 35A mutant had a greater mobility shift (33.7% of wild-type) than the PH-A/linker-A mutant. Mobility shift may not be strictly caused by phosphorylation events, and it is possible that the additional mutations somehow affect the mobility of the protein. It is also possible that any compensatory phosphorylation on the 35A mutant may affect its mobility shift, more so than the compensatory phosphorylation on the PH-A/linker-A mutant. We conclude that we have perturbed most, if not all, of the native phosphorylation sites on Cdc24, but this perturbation did not affect the function of the protein in any obvious manner.

## Discussion

In the experiments described above, we investigated the potential role for Cdc24 phosphorylation in the establishment of cell polarity and polarized growth. These experiments were motivated by the suggestion that phosphorylation plays a key role in regulating the activity and/or localization of Cdc24 to achieve symmetry breaking in a cell cycle dependent manner. Although Cdc24 phosphorylation was abundantly observed in several studies, and two kinases, Cdc28 and Cla4, were shown to phosphorylate Cdc24 *in vitro* [13], [17], the *in vivo* significance of these phosphorylation events remained unclear. Here, we have mapped over thirty *in vivo* phosphorylation sites on Cdc24. However, to our surprise, mutation of sites matching the conserved consensus sequences for the CDK and PAK family kinases has not revealed any evidence for their functional importance. We have ruled out overt effects on temperature-sensitivity, cell growth, cell morphology, protein localization, and polarization kinetics in the presence and absence of actin. Our results on the putative CDK sites are consistent with a previous study reporting that mutation of these sites did not affect Cdc24 function *in vivo* [12]. In addition, mutation of the remaining mapped sites has not revealed any functional importance in regulation of Cdc24. Many of these mutations did affect the mobility shift of Cdc24 by SDS-PAGE, indicating that we have strongly perturbed phosphorylation.

Our results for multisite phosphorylation on Cdc24 differ from results of a study reporting multisite phosphorylation of Boi1, a protein that binds Bem1 and plays an essential but redundant role in cell polarization with Boi2. In that study, the authors were able to identify phenotypes resulting from perturbation of phosphorylation. Mutation of the 12 consensus CDK sites or the 29 mapped phosphorylation sites on Boi1 resulted in temperature sensitive growth (in *Δboi2* background) and impaired Boi1 localization [32].

Several studies on proteins that are phosphorylated on multiple sites highlight the importance of the number of phosphorylation sites, as opposed to the identity of the exact residues, on a regulation mechanism. A well known example is the CDK inhibitor Sic1, which must be phosphorylated on at least six consensus CDK sites before Cdc4 can bind and target it for degradation [22]. In a more recent study, it was demonstrated that the location of the phosphorylation sites, as well as resulting overall charge of the collective phosphate groups was important for a regulation mechanism [23], [40]. It was shown that Cln/Cdc28 phosphorylates Ste5 on eight CDK sites that flank a plasma membrane binding domain. The resulting negative charges are thought to prevent the interaction of Ste5 with phospholipids of the plasma membrane. However, our results showing the lack of even a subtle defect with the *cdc24* mutant bearing 35 phosphorylation site mutations suggest that much of the phosphorylation on Cdc24 is dispensable for its function.

It is important to state that our data does not completely exclude a role for phosphorylation in Cdc24 regulation. A clear caveat is that low abundance phosphorylation sites may be overlooked in mass spectrometry analysis. It is also possible that, with further investigation, we may be able to detect a subtle polarization phenotype resulting from mutations of these sites. However, our data suggests that other modes of GEF or Cdc42 regulation may be equally or more important than Cdc24 phosphorylation, and these could mask the subtle effects of phosphorylation site mutations. For example, binding of Bud1 and Bem1 to Cdc24 has been proposed to allosterically activate Cdc24 by promoting an open conformation and/or be instrumental in bringing Cdc24 to the plasma membrane [14], [15], [19], [20]. Oligomerization of Cdc24 has also been proposed to regulate its activity [41]. Furthermore, two studies have characterized the phosphorylation of Cdc42 GTPase activating proteins (GAPs) and suggested that Cdc42 activation at the initiation of budding may be primarily regulated through downregulation of GAP activities through phosphorylation [42], [43]. Even though our study has perhaps raised more questions that it has answered, it casts doubt on the simple model that phosphorylation of Cdc24 is the key step in the establishment of cell polarity and suggests that much of protein phosphorylation in the cell could be innocent by-products of kinase activation that occur at cellular transitions.

## Materials and Methods

### Plasmid construction

All plasmids used in this study are described in Table S4. Site-directed mutagenesis of phosphorylated residues was performed using the QuikChange II XL Site-Directed Mutagenesis Kit (Stratagene), and the final product was sequenced to ensure that there were no secondary mutations introduced into the *CDC24* ORF.

### Yeast strain construction

All yeast strains used in this study are described in Table S5. Techniques for yeast cell culture and genetics were essentially as described [44]. Transformation of plasmid DNA into yeast was performed based on the lithium acetate method [45]. Transformation of PCR fragments was performed by the same method, but after transformation, cells were resuspended in non-selective media and grown for the equivalent of at least two cell cycles before plating on selective media.

Yeast strains containing mutations of the CDK or PAK consensus sites (as described in Table 1) were constructed as follows. pSW44 or its derivatives were digested with XhoI and NotI and gel purified to obtain DNA fragments containing the *CDC24* and *LEU2* ORFs. A diploid strain heterozygous for deletion of *CDC24* (RLY2775) was transformed with these fragments. Transformants were replica-plated onto media lacking leucine or YPD media containing geneticin. Transformants that were Leu^+^ and Gen^S^ (indicating that the fragment had replaced the *Δcdc24::KanMX* locus) were sporulated and dissected. Integration of the *LEU2* marker into the proper locus was confirmed by PCR. Point mutations that were successfully integrated were determined by sequencing of genomic DNA purified from the resulting strain. PCR-based GFP tagging was performed as previously described [46], [47] in order to obtain strains expressing GFP-tagged Cdc24 mutants.

Yeast strains containing the mutations of non-consensus sites (as described in Table 2) were constructed as follows. Centromeric plasmids (pSW72-73 and their derivatives) containing these mutants were transformed into a strain heterozygous for deletion of *CDC24* (RLY2775). The resulting strains were sporulated and dissected. Spores were identified that were both Leu^+^ and Gen^S^, indicating that they carry the genomic deletion of Cdc24 (*Δcdc24::KanMX*), rescued by the mutant Cdc24 on the plasmid.

### Whole cell extracts

Whole cell extracts for Western blot analysis were prepared by TCA precipitation. Cell pellets were harvested from 5–10 ml cultures grown to OD_600_ ∼0.5 and frozen in liquid nitrogen. Cell pellets were thawed, resuspended in 1 ml 20% TCA, and transferred to 1.5 ml eppendorf tubes. Cells were pelleted again by spinning for 1 min at 3000 rpm in a microcentrifuge. Glass beads (acid-washed, 425–600 µm, Sigma) and 100–200 µl of 20% TCA were added to the pellets. The mixture was then vigorously vortexed for 7 min to lyse the cells. 400 µl of 5% TCA was added to the mixture, and all of the liquid was transferred to new tubes. The protein pellet was harvested by spinning for 10 min at 3000 rpm in a microcentrifuge. After removal of liquid, the pellet was resuspended in SDS-PAGE sample buffer and titrated with Tris base if necessary.

### Western blotting

Standard methods were used for SDS-PAGE and Western blotting. Protein samples were resolved on 7.5% polyacrylamide gels, and proteins smaller than 50 kD were run off the gel in order to allow maximum separation of Cdc24 and its phosphorylated species (“smear”). Blots were developed using ECL or ECL Plus Reagents (GE Healthcare Life Sciences). For quantification, blots were scanned using the Typhoon 9400 (GE Healthcare Life Sciences) and analyzed using ImageQuantTL.

Anti-Cdc24 antiserum was raised in rabbits (Cocalico Biologicals, Reamstown, PA) using an MBP-Cdc24^472-854^ construct that was previously described [14] and generously provided by E. Bi (Univ. of Pennsylvania School of Medicine, Philadelphia, PA). Because the resulting antiserum was fairly clean (only a single cross-reacting protein was visibly detected), the antibody was not affinity purified. Anti-Arp3 antibody from goat (yG-18, to ensure equal loading of samples across lanes) and anti-myc antibody from mouse (9E10) were purchased from Santa Cruz Biotechnology.

### Purification of Cdc24-TAP

Cells were synchronized by using a temperature-sensitive *cdc28-13* allele (RLY2194). The *cdc28-13* strain was arrested in G1 at the restrictive temperature (37°C) for no more than 3 h and released into the cell cycle by cooling down the culture to 25°C using an ice water bath. Cells were monitored for bud formation and harvested about 1 h after release, when most cells were small-budded. Cells were washed once with harvest buffer (50 mM HEPES pH 7.5, 0.1 M KCl, 3 mM MgCl_2_⋅6H_2_O, 1 mM EGTA, 50 mM NaF, 120 mM β-glycerophosphate, 1 mM Na_3_VO_4_, and 1 mM DTT, supplemented with a protease inhibitor cocktail containing 0.5 mg/ml each of antipain, leupeptin, aprotinin, pepstatin, and chymostatin (Sigma)). Cell pellets were frozen in small strands in liquid nitrogen. Cell lysis was performed in a small coffee grinder (Krups), cooled with dry ice in order to keep yeast cells frozen. Lysed cells were resuspended in lysis buffer (harvest buffer brought up to high salt concentration (0.5 M KCl) and containing 1 mM PMSF). Extracts were immediately centrifuged at low speed (2430 *g*) in order to remove unlysed cells, then incubated on ice for 30 min. Extracts were then centrifuged at high speed (100,000 *g*) to remove nonsoluble components. Protein was precipitated using 40% ammonium sulfate, and the precipitate was stored at −80°C. Precipitate (which contained Cdc24-TAP) was resuspended in lysis buffer and centrifuged briefly to remove any undissolved precipitate. Extract was incubated with washed IgG Sepharose 6 Fast Flow beads (GE Healthcare Life Sciences) for 4 h at 4°C. Slurry was applied to Poly-Prep disposable columns (Bio-Rad), and beads were washed thoroughly with lysis buffer containing 0.1% NP-40. Beads were then washed with TEV buffer (harvest buffer without protease inhibitors), and incubated overnight at 4°C with purified GST-TEV (tobacco etch virus) protease to cleave off Cdc24. Beads were washed with small amount of lysis buffer, and the first two washes were pooled with the eluate. These were passed through Glutathione Sepharose 4B beads (GE Healthcare Life Sciences) to remove most of the GST-TEV protease. The flowthrough and first wash from the glutathione beads were pooled and precipitated by 20% TCA. The precipitate was resuspended in a small volume of SDS-PAGE sample buffer and resolved by SDS-PAGE on a 7.5% gel.

### Mass spectrometry analysis

Coomassie-stained bands corresponding to Cdc24 were excised from SDS-PAGE gels, destained to clarity with 50 mM ammonium bicarbonate/50% acetonitrile solution, and digested in-gel using established procedures [48]. The digested samples were extracted, dried by vacuum centrifugation, resuspended in 60 µl of 250 mM acetic acid/30% acetonitrile and treated with immobilized metal affinity chromatography (IMAC) media (Phos-Select IMAC resin, Sigma) per manufacturer's directions [49]. The IMAC resin was eluted with 50 mM K_2_HPO_4_/20% isopropanol, pH 10.5 (with 4 M ammonia in ethanol), and the two combined extracts were acidified with 5% formic acid prior to drying by vacuum centrifugation. Finally, the dried extracts were desalted using STAGE tips as described [50].

The dried, desalted samples were resuspended in 6 µl of a 1% formic acid/2.5% acetonitrile solution immediately prior to sequencing analysis. Sequencing was performed by nanoscale microcapillary LC-MS/MS essentially as described [51]. Ultimately, peptides were separated on a hand-pulled 125 µm×20 cm reverse-phase microcapillary column packed with 5 µm (200 Å) C18-AQ material as described [52]. Tandem mass spectrometry was performed on a hybrid linear (2D) ion trap – Fourier transform ion cyclotron resonance mass spectrometer over a 60 minute gradient of 5% to 32% of a 0.1% formic acid/95% acetonitrile solution as described [51]. Tandem mass spectra were de-isotoped using in-house written software and data searched using the SEQUEST algorithm with no enzyme specificity for the variable modifications+15.99491 on methionine and+79.96633 on serine, threonine and tyrosine [53], [54]. Results were initially filtered using only XCorr and mass accuracy, and candidate phosphopeptides were manually verified by inspection of the corresponding tandem mass spectra. In cases where adjacent acceptor residues precluded a definite assignment of phosphorylation locus, multiple candidate phosphorylation sites were reported.

### Serial-dilution growth assays (“spot tests”)

Overnight cultures were diluted to an OD_600_ of 0.1 to make the starting cultures. Starting cultures were pipetted into the leftmost wells of a sterile 96-well plate, and serial dilutions of four-fold were made in the subsequent wells. After thorough mixing, a sterilized frogger was used to spot the array onto the desired number of agar plates. After the cells were allowed to grow for 2–3 d at the 23°C, 30°C, 35°C, or 37°C, or 7–13 d at 16°C, plates were imaged using a digital camera or scanner. Images of each plate were processed using Adobe Photoshop CS3. The contrast was increased using the Auto Levels command, in order to better display the yeast colonies against the dark background. The images were subsequently cropped to display only the strains that were relevant to the experiment.

### Fixation and staining

Yeast cells were fixed by adding formaldehyde to a final concentration of 5% and incubating at least 2 h at room temperature with gentle shaking. Cells were then washed once in PBS, before storing at 4°C in PBS. Before scoring for morphology, fixed cultures were briefly sonicated in order to separate clumps. In order to assess bud scar patterns, Calcofluor staining was performed essentially as described [55], except that the stock solution was stored at −20°C and cells were stained with a 20- to 40-fold dilution of the Calcofluor stock solution.

### Microscopy

Widefield imaging was performed on a Nikon Eclipse E1000 fluorescent microscope with a 100X Plan-Apo TIRF NA 1.45 oil objective. Samples were illuminated with a mercury lamp through a 3-cube GFP filter, and images were acquired using a Hamamatsu ORCA-ER camera. Confocal imaging was performed on a Zeiss Axiovert 200 M inverted microscope with a 100X αPlan-Fluar NA 1.45 oil objective, attached to a Yokogawa spinning disk confocal and Hamamatsu EM-CCD C9100 camera. Images of GFP-tagged phosphorylation site mutants were collected as a series of 13 optical sections, with a step size of 0.3 µm. The final images shown are maximum *z*-projections. MetaMorph software (v. 6.3r5; Molecular Devices Corporation) was used to control both microscopes. Image J software (v. 1.37, http://rsb.info.nih.gov/ij/) was used to process the images.

### Release assays

Cells were arrested for 3 h using 5 µg/ml α-factor. In order to determine budding kinetics, cells were released into the cell cycle by washing three times in sterile water before resuspending in fresh media. Samples were taken every 10 min and fixed with formaldehyde as described above. At each time point, 100 cells were scored for budding morphology (small-budded or not). In order to determine polarization kinetics, all arrested cells were treated with 100 µM LatA (gift from P. Crews, University of California, Santa Cruz, CA) for 20 min to depolarize GFP-myc-Cdc42, and then released by washing three times in water before resuspending in fresh media with or without LatA. At each time point, >40 cells were scored for polarized localization of GFP-myc-Cdc42.

### CRIB pulldown for measurements of Cdc42^GTP^

Bacteria expressing either a GST fusion construct containing the CRIB domain from Cla4 (RLB252) or just GST for control (RLB247) were lysed using a French Press (1700 psi), and the cleared lysate was loaded onto Glutathione Sepharose 4B beads (GE Healthcare). The beads were washed and stored at 4°C until use. Yeast strains expressing GFP-myc-CDC42 (pRL369) and either wild type Cdc24 (RLY2903) or mutant Cdc24 (RLY3072) were grown up overnight. 3×10^7^ cells of each strain were spread into an even layer onto YPD plates. A total of three plates were spread for each strain and grown for 3 d at 30°C to reach stationary phase. For each strain, 1 L of YEPD media was inoculated with the cells from two plates. Half of the culture was immediately harvested. All centrifugation steps were performed at 4°C. Each pellet was washed in buffer consisting of 50 mM HEPES pH 7.5, 0.1 M KCl, 3 mM MgCl_2_⋅6H_2_O, 1 mM EGTA, 1 mM DTT, supplemented with a protease inhibitor cocktail. The cells were frozen in liquid nitrogen as small pellets and stored at −80°C. The remaining culture was incubated at 30°C and harvested as above when most of the cells had become small-budded. Yeast extracts were prepared by grinding the frozen cell pellets with a mortar and pestle that had been cooled with liquid nitrogen. Lysed yeast powder was stored at −80°C until all samples had been lysed. The powder from each sample was resuspended in a small volume of the same buffer described above, but also including 0.2% Triton X-100 and 1 mM PMSF. Extracts were spun for 30 min at 100,000 *g* to remove unlysed cells and insoluble material. The amount of protein was quantified by the Bradford Assay (Bio-Rad), and extracts were adjusted so that they had the same protein concentration. Each pull-down reaction contained 20 µl of GST-CRIB beads (or 20 µl of GST beads, diluted 1∶20 with empty beads) and 400–500 µl of yeast extract. Pull-downs were incubated at 4°C for 1 h with gentle shaking. After washing the beads 3–4 times in the same buffer, the beads were resuspended in SDS-PAGE sample buffer. All samples were boiled and spun briefly before loading onto an acrylamide gel.

## Supporting Information

Figure S1 Purification of Cdc24-TAP. A. Mobility shift of Cdc24 band changes during the cell cycle. *cdc28-13* cells (RLY434) were arrested at 37°C and synchronously released at 25°C. Samples of the culture were either fixed for morphology analysis or frozen for extract preparation every 10 min. Blots were probed with rabbit anti-Cdc24 antiserum. Faint band just above smear is a cross-reacting protein (*). B. Peak of mobility shift coincides with peak in the percentage of small-budded cells. At least 100 cells were scored for bud morphology at each timepoint. Percentage of small-budded cells was plotted over time. Budded cells present initially are likely because fixed cultures were not sonicated to separate cells before being scored. C. Comparison of Cdc24-TAP solubility in lysates prepared by either French press or manual grinding methods. Solubility was further increased by increasing salt concentration of lysis buffer. Cleared lysate (S1) was centrifuged at 10,000 *g* to give P2 and S2, and S2 was centrifuged at 100,000 *g* to give P3 and S3. Equal volumes of each fraction were loaded. Blots were probed with rabbit anti-Cdc24 antiserum. The top band of the doublet seen in some blots is a cross-reacting protein (*). D. Coomassie-stained gel showing final product of purification from RLY2194. Band at 75 kD is a heat-shock protein, and bands at 50 kD are GST and GST-TEV protease.
(0.69 MB TIF)

Figure S2 Phosphorylation site mutants do not affect localization of Cdc24. A. Confocal images of strains containing Cdc24-GFP (RLY3096, repeated from Figure 2D for reference), Cdc24^CDK-3A^-GFP (RLY3098), Cdc24^CDK-4A^-GFP (RLY3066), Cdc24^PAK-A^-GFP (RLY3065), Cdc24^CDK-DE^-GFP (RLY3100), and Cdc24^PAK-DE^-GFP (RLY3101). Scale bar represents 5 µm. B. Confocal images of strains containing Cdc24-GFP (RLY3437, repeated from Figure 5B for reference), Cdc24^PH-A^-GFP (RLY3432), Cdc24^linker-A^-GFP (RLY3434), and Cdc24^PH-A,linker-A^-GFP (RLY3430). Scale bar represents 5 µm.
(2.39 MB TIF)

Table S1 Phosphorylated Cdc24 peptides
(0.08 MB DOC)

Table S2 Ranking of phosphorylated residues
(0.01 MB PDF)

Table S3 Scansite results of phosphorylated sites
(0.06 MB PDF)

Table S4 Plasmids used in this study
(0.04 MB DOC)

Table S5 Yeast strains used in this study
(0.10 MB DOC)
