# Supplementary figures and images for: Multisite Phosphorylation of the Guanine Nucleotide Exchange Factor Cdc24 during Yeast Cell Polarization
Source: PLoS One. 2009 Aug 10;4(8):e6563. doi: 10.1371/journal.pone.0006563 (PMC2718613; doi:10.1371/journal.pone.0006563)

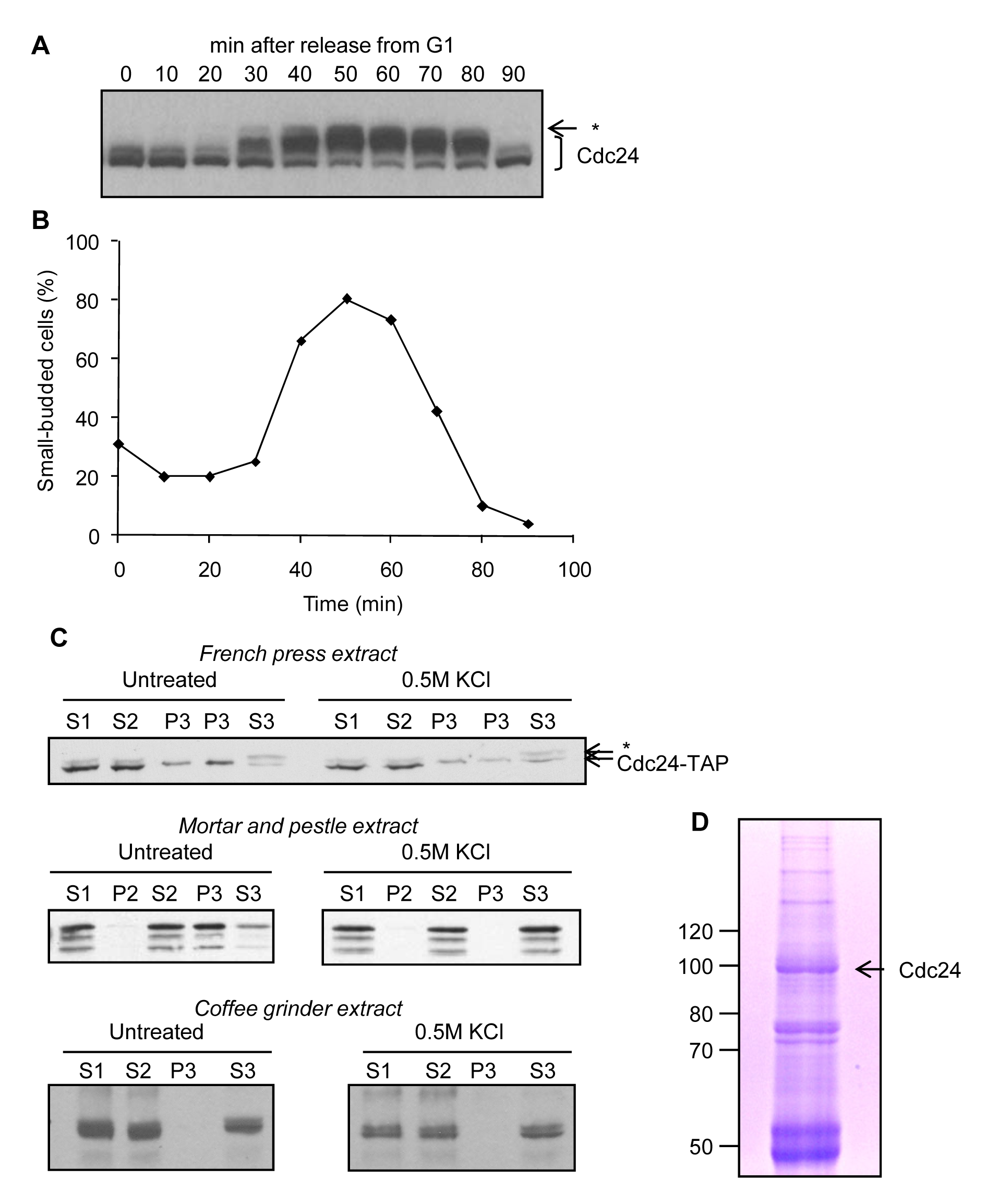

Supplement: Figure S1 — Purification of Cdc24-TAP. A. Mobility shift of Cdc24 band changes during the cell cycle. cdc28-13 cells (RLY434) were arrested at 37°C and synchronously released at 25°C. Samples of the culture were either fixed for morphology analysis or frozen for extract preparation every 10 min. Blots were probed with rabbit anti-Cdc24 antiserum. Faint band just above smear is a cross-reacting protein (*). B. Peak of mobility shift coincides with peak in the percentage of small-budded cells. At least 100 cells were scored for bud morphology at each timepoint. Percentage of small-budded cells was plotted over time. Budded cells present initially are likely because fixed cultures were not sonicated to separate cells before being scored. C. Comparison of Cdc24-TAP solubility in lysates prepared by either French press or manual grinding methods. Solubility was further increased by increasing salt concentration of lysis buffer. Cleared lysate (S1) was centrifuged at 10,000 g to give P2 and S2, and S2 was centrifuged at 100,000 g to give P3 and S3. Equal volumes of each fraction were loaded. Blots were probed with rabbit anti-Cdc24 antiserum. The top band of the doublet seen in some blots is a cross-reacting protein (*). D. Coomassie-stained gel showing final product of purification from RLY2194. Band at 75 kD is a heat-shock protein, and bands at 50 kD are GST and GST-TEV protease. (0.69 MB TIF) [file pone.0006563.s001.tif]

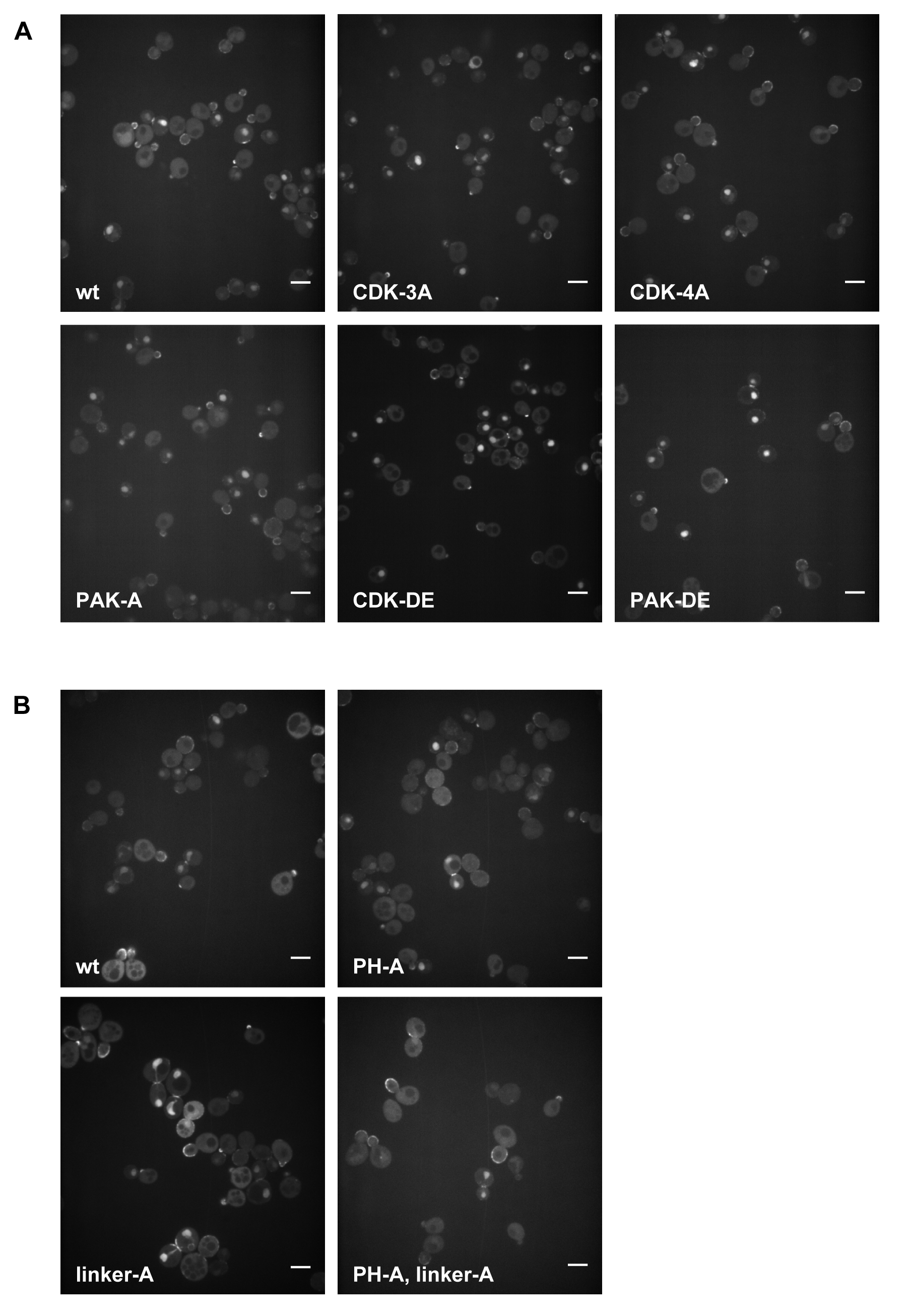

Supplement: Figure S2 — Phosphorylation site mutants do not affect localization of Cdc24. A. Confocal images of strains containing Cdc24-GFP (RLY3096, repeated from Figure 2D for reference), Cdc24CDK-3A-GFP (RLY3098), Cdc24CDK-4A-GFP (RLY3066), Cdc24PAK-A-GFP (RLY3065), Cdc24CDK-DE-GFP (RLY3100), and Cdc24PAK-DE-GFP (RLY3101). Scale bar represents 5 µm. B. Confocal images of strains containing Cdc24-GFP (RLY3437, repeated from Figure 5B for reference), Cdc24PH-A-GFP (RLY3432), Cdc24linker-A-GFP (RLY3434), and Cdc24PH-A,linker-A-GFP (RLY3430). Scale bar represents 5 µm. (2.39 MB TIF) [file pone.0006563.s002.tif]
