# Supplementary material for: Multisite Phosphorylation of the Guanine Nucleotide Exchange Factor Cdc24 during Yeast Cell Polarization
Source: PLoS One. 2009 Aug 10;4(8):e6563. doi: 10.1371/journal.pone.0006563 (PMC2718613; doi:10.1371/journal.pone.0006563)
Supplement: Table S1 — Phosphorylated Cdc24 peptides (0.08 MB DOC) [file pone.0006563.s003.doc]

Table S1. Phosphorylated Cdc24 peptides

| **Peptide** | **Amino acids** | **Peptide Count** | **Residues** |
| --- | --- | --- | --- |
| R.FAS*GTS*LSDLKPK.P | 7-19 | 3 | 9-14 |
| R.FAS*GTSLS*DLKPK.P | 7-19 | 1 | 9-14 |
| R.FAS*GTSLSDLKPK.P | 7-19 | 5 | 9-14 |
| R.FASGT*SLSDLKPK.P | 7-19 | 1 | 9-14 |
| R.FASGTS*LSDLKPK.P | 7-19 | 5 | 9-14 |
| R.FAS*GTS*LSDLKPKPS.A | 7-21 | 1 | 9-14 |
| R.FAS*GTSLSDLKPKPS.A | 7-21 | 1 | 9-14 |
| R.FAS*GTS*LSDLKPKPSAT.S | 7-23 | 1 | 9-14 |
| R.FASGT*SLSDLKPKPSAT.S | 7-23 | 1 | 9-14 |
| R.FASGTS*LSDLKPKPSAT.S | 7-23 | 1 | 9-14 |
| K.SNGANRDS*SDLAPTLR.S | 93-108 | 3 | 100-101 |
| K.SNGANRDSS*DLAPTLR.S | 93-108 | 1 | 100-101 |
| N.GANRDS*SDLAPTLR.S | 95-108 | 1 | 100-101 |
| N.RDS*SDLAPTLR.S | 98-108 | 1 | 100-101 |
| R.DS*SDLAPTLR.S | 99-108 | 1 | 100-101 |
| R.DSSDLAPT*LR.S | 99-108 | 1 | 106 |
| K.KS*ASSLILK.K | 525-533 | 2 | 526-529 |
| K.KSASS*LILK.K | 525-533 | 1 | 526-529 |
| K.S*ASS*LILK.K | 526-533 | 1 | 526-529 |
| K.S*ASSLILK.K | 526-533 | 1 | 526-529 |
| K.SAS*SLILK.K | 526-533 | 1 | 526-529 |
| K.S*ASSLILKK.K | 526-534 | 1 | 526-529 |
| K.KSSTS*ASISASNITDNNGS*PHHSYHK.R | 535-560 | 1 | 539, 553 |
| K.KSSTSASISASNITDNNGS*PHHSYHK.R | 535-560 | 1 | 553 |
| K.SSTSASISASNITDNNGS*PH.H | 536-555 | 2 | 553 |
| K.SSTSASISASNITDNNGS*PHHSYH.K | 536-559 | 1 | 553 |
| K.SSTSASISASNITDNNGSPHHSY*H.K | 536-559 | 1 | 557-558 |
| K.SSTSASISASNITDNNGS*PHHS*YHK.R | 536-560 | 2 | 553, 557-558 |
| K.SSTSASISASNITDNNGS*PHHSYHK.R | 536-560 | 5 | 553 |
| K.SSTSASISASNITDNNGSPHHSY*HK.R | 536-560 | 1 | 557-558 |
| K.SSTSASISASNITDNNGS*PHHS*YHKR.H | 536-561 | 1 | 553, 557-558 |
| K.RHS*NSSSSNNIHLSSS.S | 561-576 | 1 | 563 |
| R.HS*NSSSSNNIHLSSSSAAAIIHSSTN.S | 562-587 | 1 | 563 |
| R.HSNS*SSS*NNIHLSSSSAAAIIHSSTN.S | 562-587 | 1 | 565-568 |
| N.SSDNNSNNSS*SSSLFK.L | 588-603 | 1 | 596-600 |
| N.NSNNS*SSSSLFK.L | 592-603 | 1 | 596-600 |
| N.SNNS*SSSSLFK.L | 593-603 | 1 | 596-600 |
| K.SSSMM#S*PTTTM#NTPNHHNSR.Q | 692-711 | 1 | 697 |
| K.RVS*DVLPK.R | 727-734 | 3 | 729 |
| R.VS*DVLPK.R | 728-734 | 1 | 729 |
| K.RRT*TS*SS*FESEIK.S | 735-747 | 1 | 737-741 |
| K.RRT*TSSS*FESEIK.S | 735-747 | 2 | 737-741 |
| K.RRT*TSSSFESEIK.S | 735-747 | 1 | 737-741 |
| K.RRTT*SSS*FESEIK.S | 735-747 | 1 | 737-741 |
| R.RTT*SSS*FESEIK.S | 736-747 | 2 | 737-741 |
| R.RTT*SSSFESEIK.S | 736-747 | 4 | 737-741 |
| R.RTTS*SSFESEIK.S | 736-747 | 1 | 737-741 |
| R.RTTSSS*FESEIK.S | 736-747 | 1 | 737-741 |
| R.TTSSSFESEIKS*ISENFK.N | 736-754 | 1 | 748 |
| R.T*TSSS*FESEIK.S | 737-747 | 1 | 737-741 |
| R.T*TSSSFESEIK.S | 737-747 | 1 | 737-741 |
| R.TTS*SS*FESEIK.S | 737-747 | 3 | 737-741 |
| R.TTSSS*FESEIK.S | 737-747 | 4 | 737-741 |
| R.TTS*SSFESEIKS*ISENFK.N | 737-754 | 1 | 737-741, 748 |
| T.SSS*FESEIK.S | 739-747 | 1 | 737-741 |
| K.SIS*ENFK.N | 748-754 | 1 | 750 |
| K.SISENFKNS*IPESSILFR.I | 748-765 | 1 | 756 |
| K.ISNTHNNNIS*PITK.I | 802-815 | 1 | 811 |
