# Supplementary material for: Multisite Phosphorylation of the Guanine Nucleotide Exchange Factor Cdc24 during Yeast Cell Polarization
Source: PLoS One. 2009 Aug 10;4(8):e6563. doi: 10.1371/journal.pone.0006563 (PMC2718613; doi:10.1371/journal.pone.0006563)
Supplement: Table S2 — Ranking of phosphorylated residues (0.01 MB PDF) [file pone.0006563.s004.pdf]

Table S2. Ranking of phosphorylated residues

Peptides	Residues	No. of Residues	Peptides/no. of residues	NetPhos	NetPhos (average)	Scansite (smallest)	Rank (peptides/no. of residues)	Rank (NetPhos)	Rank (Scansite)	Total Rank
24	T737, T738, S739, S740, S741	5	4.8	0.638, 0.989, 0.987, 0.990, 0.998	0.918	0.17	3	6	1	10
13	S553	1	13.0	0.908	0.908	0.4	1	8	2	11
2	S563	1	2.0	0.995	0.995	1.6	8	1	5	14
4	S729	1	4.0	0.726	0.726	0.93	4	12	4	20
2	S748	1	2.0	0.989	0.989		8	2	11	21
1	S697	1	1.0	0.985	0.985		9	3	11	23
1	S756	1	1.0	0.961	0.961	4.8	9	4	10	23
7	S100, S101	2	3.5	0.851, 0.967	0.909		5	7	11	23
7	S526, S528	3	2.3	0.376, 0.709, 0.852	0.646	0.7	7	13	3	23
1	S539	1	1.0	0.954	0.954		9	5	11	25
1	S565, S566, S567, S568	4	0.3	0.633, 0.980	0.807	2.1	11	9	7	27
20	S009, T011, S012, S014	4	5.0	0.593, 0.117, 0.994, 0.195	0.475		2	14	11	27
3	S596, S597, S598, S599, S600	5	0.6	0.794	0.794	2.2	10	10	8	28
1	T106	1	1.0	0.737	0.737	4.1	9	11	9	29
5	S557, Y558	2	2.5	0.433, 0.052	0.243		6	15	11	32
1	S750	1	1.0	0.019	0.019	2	9	17	6	32
1	S811	1	1.0	0.027	0.027		9	16	11	36
