# Supplementary material for: Multisite Phosphorylation of the Guanine Nucleotide Exchange Factor Cdc24 during Yeast Cell Polarization
Source: PLoS One. 2009 Aug 10;4(8):e6563. doi: 10.1371/journal.pone.0006563 (PMC2718613; doi:10.1371/journal.pone.0006563)
Supplement: Table S3 — Scansite results of phosphorylated sites (0.06 MB PDF) [file pone.0006563.s005.pdf]

Table S3. Scansite results of phosphorylated sites

Peptides	Residues	Motif	Percentile rank	SA
20	S009			
	T011			
	S012			
	S014			
7	S100			
	S101			
1	T106	PKC α/β/γ	4.098%	1.118
7	S526	PKC μ	2.467%	2.228
	S528			
	S529	PKC α/β/γ	0.701%	0.198
		CaM-kinase 2	1.003%	
		Casein kinase 1	4.578%	
1	S539			
13	S553	Cdk5	0.423%	1.453
		Cdc2	0.478%	
5	S557			
	Y558			
2	S563	PKA	1.631%	2.455
1	S565			
	S566			
	S567			
	S568	Casein kinase 1	2.131%	1.078
3	S596			
	S597			
	S598			
	S599			
	S600	PKC ε	3.289%	0.788
		PKC δ	3.849%	
		Casein kinase 1	2.205%	
1	S697			
4	S729	PKA	0.929%	0.456
24	T737	PKA	0.707%	3.288
		PKC ε	3.428%	
	T738	CaM-kinase 2	0.170%	2.250
		PKA	0.733%	
		PKC ε	4.789%	
	S739	14-3-3 Mode 1	1.835%	0.995
	S740	PKC ε	0.190%	1.194
		PKC α/β/γ	4.252%	
	S741	PKC ε	0.828%	1.108
		PKC δ	0.815%	
		PKC α/β/γ	1.554%	
		DNA PK	0.749%	
2	S748			
1	S750	14-3-3 Mode 1	2.042%	0.722
1	S756	PKC ε	4.789%	1.925
1	S811			
