# Supplementary material for: Multisite Phosphorylation of the Guanine Nucleotide Exchange Factor Cdc24 during Yeast Cell Polarization
Source: PLoS One. 2009 Aug 10;4(8):e6563. doi: 10.1371/journal.pone.0006563 (PMC2718613; doi:10.1371/journal.pone.0006563)
Supplement: Table S4 — Plasmids used in this study (0.04 MB DOC) [file pone.0006563.s006.doc]

Table S4. Plasmids used in this study

| **Plasmid** | **Description** |
| --- | --- |
| pRL369 | pCDC42-GFP-myc_6_-CDC42 in pRS306 |
| pSW44 | Bluescript vector containing CDC24 with 500bp of 3’UTR, LEU2 inserted in HpaI site within UTR |
| pSW46 | Derivative of pSW44 with CDK-3A mutations |
| pSW47 | Derivative of pSW44 with PAK-A mutations |
| pSW52 | Derivative of pSW44 with CDK-6A mutations |
| pSW56 | Derivative of pSW44 with CDK-A, PAK-A mutations |
| pSW57 | Derivative of pSW44 with CDK-DE mutations |
| pSW58 | Derivative of pSW44 with PAK-DE mutations |
| pSW72 | pRS315 vector (LEU/CEN) containing CDC24-GFP |
| pSW73 | pRS315 vector (LEU/CEN) containing CDC24 with 500bp of 3’UTR |
| pSW76 | Derivative of pSW72 with PH-A/linker-A mutations |
| pSW77 | Derivative of pSW73 with PH-A/linker-A mutations |
| pSW78 | Derivative of pSW72 with PH-A mutations |
| pSW79 | Derivative of pSW73 with PH-A mutations |
| pSW80 | Derivative of pSW72 with linker-A mutations |
| pSW81 | Derivative of pSW73 with linker-A mutations |
| pSW86 | Derivative of pSW73 with 35A mutations |
| pSW87 | Derivative of pSW72 with 35A mutations |
