# Supplementary material for: Multisite Phosphorylation of the Guanine Nucleotide Exchange Factor Cdc24 during Yeast Cell Polarization
Source: PLoS One. 2009 Aug 10;4(8):e6563. doi: 10.1371/journal.pone.0006563 (PMC2718613; doi:10.1371/journal.pone.0006563)
Supplement: Table S5 — Yeast strains used in this study (0.10 MB DOC) [file pone.0006563.s007.doc]

Table S5. Yeast strains used in this study

| **Strain** | **Genotype** | **Source** |
| --- | --- | --- |
| 171 | Mat**a** ura3 leu2 cdc42-1 | R. Li yeast collection |
| 434 | Mat**a** leu2 trp1 ura3 his3 cdc28-13  (W303 background) | R. Li yeast collection |
| 2194 | Mat**a** leu2 trp1 ura3 his3 cdc28-13  CDC24-TAP::URA3 (W303 background) | This study |
| 2530 | Mat**a** his3Δ1 leu2Δ0 met15Δ0 ura3Δ0 | Yeast Knockout Collection  (BY4741) |
| 2771 | Mat**a** his3Δ1 leu2Δ0 ura3Δ0 Δbem1::KanMX | Yeast Knockout Collection |
| 2773 | Mat**a** his3Δ1 leu2Δ0 ura3Δ0 Δbud1::KanMX | Yeast Knockout Collection |
| 2775 | Mat**a**/α his3Δ1/ leu2Δ0/ met15Δ0/+ lys2Δ0/+ ura3Δ0/ Δcdc24::KanMX/+ | Yeast Knockout Collection |
| 2848 | Mat**a**/α his3Δ1/ leu2Δ0/ met15Δ0/+ lys2Δ0/+ ura3Δ0/ CDC24::LEU2/+ | This study |
| 2849 | Mat**a**/α his3Δ1/ leu2Δ0/ met15Δ0/+ lys2Δ0/+ ura3Δ0/ CDC24^CDK-3A^::LEU2/+ | This study |
| 2850 | Mat**a**/α his3Δ1/ leu2Δ0/ met15Δ0/+ lys2Δ0/+ ura3Δ0/ CDC24^PAK-A^::LEU2/+ | This study |
| 2853 | Mat**a** his3Δ1 leu2Δ0 ura3Δ0 CDC24::LEU2 | This study |
| 2855 | Mat**a** his3Δ1 leu2Δ0 ura3Δ0  CDC24^CDK-3A^::LEU2 | This study |
| 2858 | Mat**a** his3Δ1 leu2Δ0 ura3Δ0  CDC24^PAK-A^::LEU2 | This study |
| 2903 | Mat**a** his3Δ1 leu2Δ0 ura3Δ0 CDC24::LEU2  pRL369 (pCDC42-GFP-myc_6_-CDC42 / pRS306) | This study |
| 2921 | Mat**a**/α his3Δ1/ leu2Δ0/ ura3Δ0/  CDC24^CDK-4A^::LEU2/+ | This study |
| 2924 | Mat**a**/α his3Δ1/ leu2Δ0/ ura3Δ0/  CDC24^CDK-A,PAK-A^::LEU2/+ | This study |
| 2925 | Mat**a**/α his3Δ1/ leu2Δ0/ ura3Δ0/  CDC24^CDK-DE^::LEU2/+ | This study |
| 2926 | Mat**a**/α his3Δ1/ leu2Δ0/ ura3Δ0/  CDC24^PAK-DE^::LEU2/+ | This study |
| 2934 | Mat**a** his3Δ1 leu2Δ0 ura3Δ0  CDC24^CDK-4A^::LEU2 | This study |
| 2937 | Matα his3Δ1 leu2Δ0 ura3Δ0  CDC24^CDK-A, PAK-A^::LEU2 | This study |
| 2938 | Mat**a** his3Δ1 leu2Δ0 ura3Δ0  CDC24^CDK-DE^::LEU2 | This study |
| 3065 | Mat**a** his3Δ1 leu2Δ0 ura3Δ0  CDC24^PAK-A^-GFP::HIS3::LEU2 | This study |
| 3066 | Mat**a** his3Δ1 leu2Δ0 ura3Δ0  CDC24^CDK-4A^-GFP::HIS3::LEU2 | This study |
| 3072 | Mat**a** his3Δ1 leu2Δ0 ura3Δ0  CDC24^CDK-A, PAK-A^::LEU2  pRL369 (pCDC42-GFP-myc6-CDC42 / pRS306) | This study |
| 3088 | Mat**a** his3Δ1 leu2Δ0 ura3Δ0  CDC24^CDK-A, PAK-A^::LEU2 | This study |
| 3089 | Mat**a** his3Δ1 leu2Δ0 ura3Δ0  CDC24^PAK-DE^::LEU2 | This study |
| 3093 | Mat**a**/α his3Δ1/ leu2Δ0/ ura3Δ0/  CDC24^CDK-A, PAK-A^::LEU2/+ Δbud1::KanMX/+ | This study |
| 3095 | Mat**a**/α his3Δ1/+ leu2Δ0/leu2 ura3Δ0/ura3  CDC24^CDK-A, PAK-A^::LEU2/+ cdc42-1/+ | This study |
| 3096 | Mat**a** his3Δ1 leu2Δ0 ura3Δ0  CDC24-GFP::HIS3::LEU2 | This study |
| 3098 | Mat**a** his3Δ1 leu2Δ0 ura3Δ0  CDC24^CDK-3A^-GFP::HIS3::LEU2 | This study |
| 3099 | Mat**a** his3Δ1 leu2Δ0 ura3Δ0  CDC24^CDK-A,PAK-A^-GFP::HIS3::LEU2 | This study |
| 3100 | Mat**a** his3Δ1 leu2Δ0 ura3Δ0  CDC24^CDK-DE^-GFP::HIS3::LEU2 | This study |
| 3101 | Mat**a** his3Δ1 leu2Δ0 ura3Δ0  CDC24^PAK-DE^-GFP::HIS3::LEU2 | This study |
| 3102 | Mat**a**/α his3Δ1/ leu2Δ0/ ura3Δ0/  CDC24^CDK-A, PAK-A^::LEU2/+ Δbem1::KanMX/+ | This study |
| 3353 | Mat**a**/α his3Δ1/ leu2Δ0/ ura3Δ0/  Δcdc24::KanMX/+  pSW76 (CDC24^PH-A, linker-A^-GFP/pRS315) | This study |
| 3354 | Mat**a**/α his3Δ1/ leu2Δ0/ ura3Δ0/  Δcdc24::KanMX/+  pSW77 (CDC24^PH-A, linker-A^/pRS315) | This study |
| 3355 | Mat**a**/α his3Δ1/ leu2Δ0/ ura3Δ0/  Δcdc24::KanMX/+  pSW78 (CDC24^PH-A^-GFP/pRS315) | This study |
| 3356 | Mat**a**/α his3Δ1/ leu2Δ0/ ura3Δ0/  Δcdc24::KanMX/+  pSW79 (CDC24^PH-A^/pRS315) | This study |
| 3357 | Mat**a**/α his3Δ1/ leu2Δ0/ ura3Δ0/  Δcdc24::KanMX/+  pSW80 (CDC24^linker-A^-GFP/pRS315) | This study |
| 3358 | Mat**a**/α his3Δ1/ leu2Δ0/ ura3Δ0/  Δcdc24::KanMX/+  pSW81 (CDC24^linker-A^/pRS315) | This study |
| 3371 | Mat**a**/α his3Δ1/ leu2Δ0/ ura3Δ0/  Δcdc24::KanMX/+  pSW72 (CDC24-GFP/pRS315) | This study |
| 3372 | Mat**a**/α his3Δ1/ leu2Δ0/ ura3Δ0/  Δcdc24::KanMX/+  pSW73 (CDC24/pRS315) | This study |
| 3390 | Mat**a** his3Δ1 leu2Δ0 ura3Δ0  Δcdc24::KanMX  pSW77 (CDC24^PH-A, linker-A^/pRS315) | This study |
| 3391 | Mat**a** his3Δ1 leu2Δ0 ura3Δ0  Δcdc24::KanMX  pSW79 (CDC24^PH-A^/pRS315) | This study |
| 3393 | Mat**a** his3Δ1 leu2Δ0 ura3Δ0  Δcdc24::KanMX  pSW81 (CDC24^linker-A^/pRS315) | This study |
| 3395 | Mat**a** his3Δ1 leu2Δ0 ura3Δ0  Δcdc24::KanMX  pSW73 (CDC24/pRS315) | This study |
| 3430 | Mat**a** his3Δ1 leu2Δ0 ura3Δ0  Δcdc24::KanMX  pSW76 (CDC24^PH-A, linker-A^-GFP/pRS315) | This study |
| 3432 | Mat**a** his3Δ1 leu2Δ0 ura3Δ0  Δcdc24::KanMX  pSW78 (CDC24^PH-A^-GFP/pRS315) | This study |
| 3434 | Mat**a** his3Δ1 leu2Δ0 ura3Δ0  Δcdc24::KanMX  pSW80 (CDC24^linker-A^-GFP/pRS315) | This study |
| 3437 | Mat**a** his3Δ1 leu2Δ0 ura3Δ0  Δcdc24::KanMX  pSW72 (CDC24-GFP/pRS315) | This study |
| 3454 | Mat**a**/α his3Δ1/ leu2Δ0/ ura3Δ0/  Δcdc24::KanMX/+  pSW86 (CDC24^35A^/pRS315) | This study |
| 3457 | Mat**a**/α his3Δ1/ leu2Δ0/ ura3Δ0/  Δcdc24::KanMX/+  pSW87 (CDC24^35A^-GFP/pRS315) | This study |
| 3461 | Mat**a** his3Δ1 leu2Δ0 ura3Δ0  Δcdc24::KanMX  pSW86 (CDC24^35A^/pRS315) | This study |
| 3468 | Mat**a** his3Δ1 leu2Δ0 ura3Δ0  Δcdc24::KanMX  pSW87 (CDC24^35A^-GFP/pRS315) | This study |

Strains are in the s288c genetic background unless otherwise noted.
